# Comparison of stereotactic brain biopsy techniques in dogs: neuronavigation, 3D-printed guides, and neuronavigation with 3D-printed guides

**DOI:** 10.3389/fvets.2024.1406928

**Published:** 2024-06-10

**Authors:** Richard L. Shinn, Christopher Hollingsworth, Rell L. Parker, John H. Rossmeisl, Stephen R. Werre

**Affiliations:** ^1^Clinical Applications Laboratory, Department of Small Animal Clinical Sciences, Virginia Maryland College of Veterinary Medicine, Virginia Tech, Blacksburg, VA, United States; ^2^Department of Cancer Biology, Wake Forest Baptist Comprehensive Cancer Center, Winston-Salem, NC, United States; ^3^Department of Population Health Sciences, Virginia Maryland College of Veterinary Medicine, Virginia Tech, Blacksburg, VA, United States

**Keywords:** stereotactic brain biopsy, neuronavigation, Brainsight, 3D-printed guide, computed tomography

## Abstract

The objective of this research was to compare two previously described stereotactic brain biopsy (SBB) techniques, three-dimensional skull contoured guides (3D-SCGs) and neuronavigation with Brainsight, to a novel SBB technique using Brainsight combined with a 3D-printed headframe (BS3D-HF) to improve the workflow of SBB in dogs. This was a prospective methods comparison with five canine cadavers of different breeds and size. Initial helical CT was performed on cadavers with fiducial markers in place. Ten different target points were randomly selected for each method. The headframe for the BS3D-HF was designed and printed. Trajectories were planned for each method. Steinmann pins (SPs) were placed into the target points using the planned trajectories for each method, and CT was repeated (post CT). Accuracy was assessed by overlaying the initial CT onto the post CT and measuring the difference of the planned target point to the SP placement. For 3D-SCG, the median deviation was 2.48 mm (0.64–4.04). With neuronavigation, the median deviation was 3.28 mm (1.04–4.64). For BS3D-HF, the median deviation was 14.8 mm (8.87–22.1). There was no significant difference between 3D-SCG and neuronavigation for the median deviation (*p* = 0.42). When comparing BS3D-HF to 3D-SCG, there was a significant difference in the median deviation (*p* < 0.0001). Additionally, when comparing BS3D-HF to neuronavigation, there was a significant difference for the median deviation (*p* < 0.0001). Our findings concluded that both 3D-SCGs and neuronavigation were accurate for SBB, however BS3D-HF was not. Although feasible, the current BS3D-HF technique requires further refinement before it can be recommended for use for SBB in dogs.

## 1 Introduction

**Figure 1:**
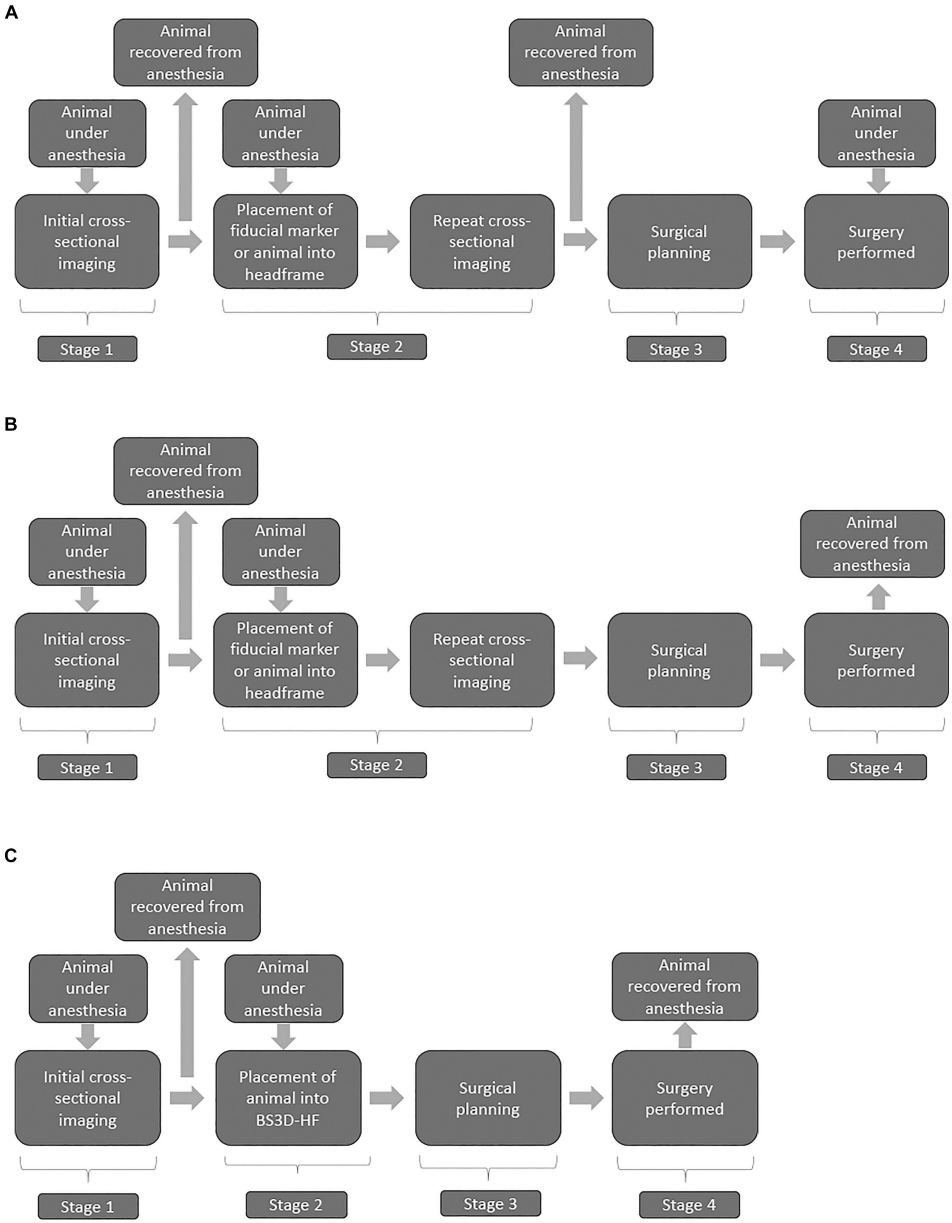
Flow charts illustrating three stage **(A)** two stage **(B)**, and BS3D-HF abbreviated two-stage **(C)** stereotactic brain biopsy (SBB) workflows. Stage 1 is the initial diagnosis of a brain lesion found on MRI and/or CT. Stage 2 places the animal into the SBB equipment, generally either a headframe or fiducial markers followed by repeated CT for most workflows; however, MRI is sometimes performed. This stage requires a second anesthesia for the animal, and potentially surgical placement of fiducial markers. The use of 3D-SCG or BS3D-HF in workflows allow bypassing f repeated cross-sectional imaging in stage 2. Stage 3 is the surgical planning, where the target point is selected along with the optimal trajectory. Recovery from anesthesia is not required at this stage, but in general the timeline does not allow for Stage 2 to immediately flow into Stage 3 with the animal still under anesthesia. Stage 4 is when the SBB is performed. Some animal holding devices at this stage can be intrusive requiring additional skin and muscle incisions. With stage 4, due to registration errors, brain lesion alterations, or unexpected animal complications (e.g., blood vessel not seen on CT), surgical refinement might be needed.

Both magnetic resonance imaging (MRI) and computed tomography (CT) have become readily available in veterinary practice and are critical for establishing a diagnosis in dogs with intracranial disease (1). Although sensitivity for detection of intracranial lesions is high with advanced imaging, specificity is limited (2). For diagnosis of an intracranial lesion, often biopsy is needed, ideally with stereotactic brain biopsy (SBB) (2). Over a dozen different publications have discussed various SBB techniques in veterinary medicine, each with their own advantages and disadvantages (2–18). These techniques can be grouped into two main categories, frame-based and frameless, with the latter often referred to as neuronavigation (16, 19, 20). With both frame-based and frameless techniques, workflow comprises four main stages: (1) initial cross-sectional imaging, (2) cross-sectional imaging including the stereotactic device, (3) surgical planning, and (4) surgical refinement (Figure 1) (20). Although this workflow is the standard of care for stereotactic surgery, it has several drawbacks and limitations. The first major limitation is that stereotactic biopsy procedures require multiple anesthetic episodes to complete the workflow. While multi-staged workflows could subject the animal to as many as three separate anesthetic events (Figure 1A), other clinically utilized stereotactic biopsy procedures require two anesthetic episodes by performing stages 2–4 in a single anesthetic (Figure 1B) event (2, 7, 15). The workflow also requires repeating advanced imaging (21). In people this is of concern due to the increased radiation exposure with CT, although this is not as much of a concern with animals. Depending on the specific stereotactic system being used, placement of fiducial markers or headframe could require additional surgery. Third, the headframe or fiducial markers could cause imaging artifacts which could interfere with surgical planning (Supplementary Figure S1). Finally, registration errors can occur with movement of the frame, the fiducial markers, or the animal within the frame.

The use of three-dimensional (3D) printed guides can bypass the need for repeating cross-sectional imaging in stage 2 (Figure 1C) of the workflow (9). The downside of 3D printed guides occurs in stage 4, as intraoperative refinement is not possible as the 3D printed guides are contoured to the shape of the bone (9, 14). This is where frameless SBB excels, but it is not possible to bypass repeating imaging in stage 2 in the workflow with neuronavigation (Figure 1B) (20). Another downside of some neuronavigation platforms is that the animal has to be placed in a surgical head clamp which is cumbersome and requires additional incisions. Frame-based SBB techniques have the disadvantage of not being able to bypass stage 2 and intraoperative refinement is limited. To overcome the disadvantages of each technique, our group designed a technique which combines neuronavigation (Brainsight^TM^) (RRID:SCR_009539) with a 3D printed headframe (BS3D-HF). Such a technique has never been investigated in people or in the veterinary English literature. Our primary aim was to assess the feasibility of BS3D-HF. We hypothesized that target trajectory using BS3D-HF can be designed and Steinmann pins placed based on the initial CT (Figure 1). Our secondary aim was to compare the accuracy of BS3D-HF to both traditional neuronavigation with fiducial markers (6), and 3D skull contoured guides (3D-SCGs) (9). We hypothesized that the BS3D-HF would have superior accuracy when compared to traditional neuronavigation or 3D-SCGs.

## 2 Materials and methods

### 2.1 Subjects

In this prospective methods comparison study, five canine cadavers of various breeds and sizes were used. All procedures performed in this study were approved by the institutional animal care and use committee (23-071). The accuracy of 3D-SCG and neuronavigation has been previously published, with methods used to assess the needle placement error described by our group previously (6, 9). In previous publications, the mean needle placement error for neuronavigation was 1.79 ± 0.87 mm (6). For 3D-SCGs, the mean needle placement error was 2.7 ± 1.1 mm (9). Therefore, we performed a power analysis to determine the necessary sample size with an alpha of 0.05 and power of 0.80, a total of 7 different needle placements would be needed in each group, however we increased this value to 10 to help improve statistical power.

### 2.2 Fiducial marker

**Figure 2:**
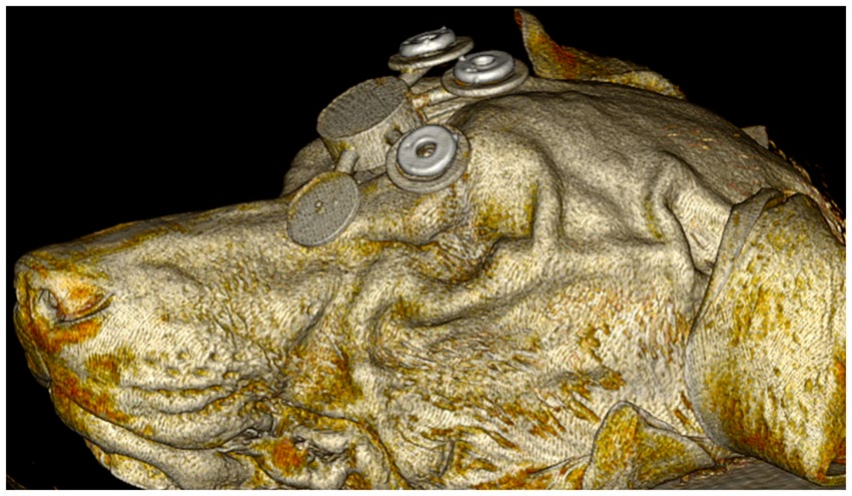
Initial computed tomography rendering of dog 1 with the 3D fiducial marker holder fixed to the rostromedial portion of the outer table of the frontal bone with acrylic screws (not shown).

For each dog, a 3D printed fiducial marker holder was designed using the 3D modeling software Autodesk^®^ Meshmixer (RRID:SCR_015736) (V3.5.474, San Rafael, CA, United States), as shown in Figure 2. The design was then exported as an STL file and uploaded into Formlabs^®^ PreForm (V3.3, Somerville, MA, United States) 3D slicing software. Printing thickness was set to 0.1 mm and the auto-setup print function was used for printing. Files were then exported to the Formlabs 3B+ printer (Formlabs, Somerville, MA) and printed using the Surgical Guide resin (RS-F2-SGAM-01). Fiducial marker holders were then washed in 99% isopropyl alcohol for 20 min, then placed in the Form Cure UV station for 2 h to maximize the resin properties. Following the UV cure, three multimodal fiducial markers (IZI Medical) were placed on each fiducial marker holder (Figure 2). The fiducial marker holders were then fixed to the rostromedial portion of the outer table of the frontal bone with 2 mm × 10 mm acrylic screws. A linear skin incision was made on midline over the frontal bone where the fiducial marker holder would be placed. A 1.5 mm drill bit was used to pre-drill then the guide was fixed with the acrylic screws.

### 2.3 Initial CT

Helical CT (Toshiba Aquilion 64-slice CT scanner, Japan) was performed on each of the 5 cadavers with the fiducial marker holder attached using the following parameters: field of view (FOV) was set at 512 × 512, slice thickness was set at 0.5 mm in transverse plane, cadavers were sternal, and a bone reconstruction algorithm was used. Images were transferred using Digital Imaging and Communications in Medicine (DICOM) format to a secondary workstation using open-source image viewing medical software (Horos-64 bit version 3.3.6, Annapolis, MS, United States) for target point planning and for creating STL files. Both skin and skull STL files were exported for each dog using the Grow Region and 3D Surface Rendering tools within the software. Fiducial marker holders were removed and the cadavers were then placed in a cooler (Darwin Chambers, Lab-G1NN, 40°C) during target point planning for one week.

### 2.4 Target point planning

Target points that were included for possible sampling were the right and left caudate nucleus, hippocampus, piriform lobe, occipital lobe corona radiata, thalamus (region of the ventral rostral nucleus) and midbrain (within the tegmentum near the region of the red nucleus), meaning a total of 60 initial different target points. Ten different target points were assigned for each of the three methods, or a total of 30 different target points, and were randomly assigned using Microsoft Excel. Once the target point was assigned, a 3 × 3 × 1.5 mm sphere (height, width, depth) region of interest (ROI) was created at the target point.

### 2.5 Creation of the BS3D-HF headframe

**Figure 3:**
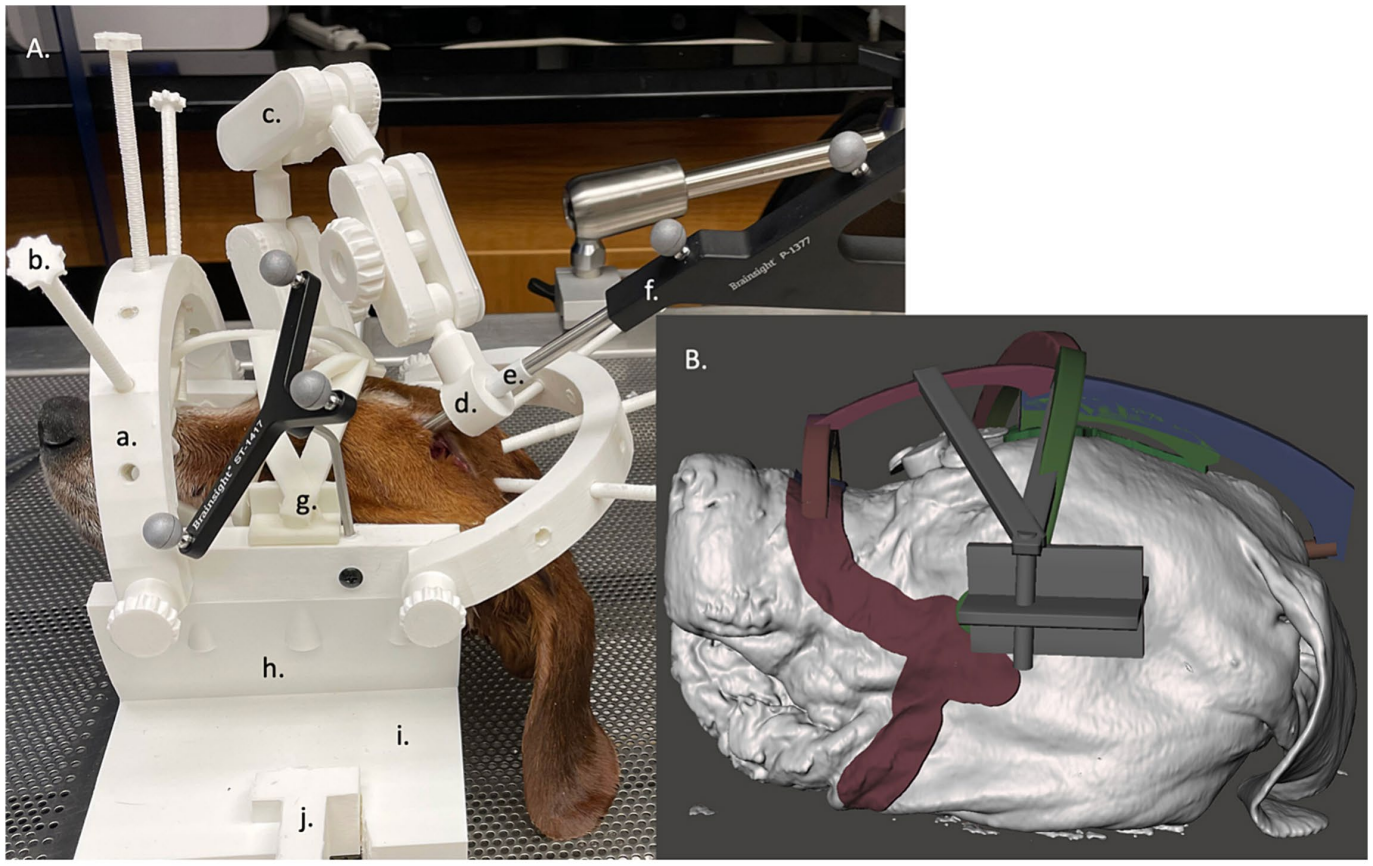
**(A)** Neuronavigation combined with a 3D-printed headframe (BS3D-HF) with cadaver placed in the face mask. The headframes consisted of five major components: the base which secured to the operating table (i) with a table clamp (j), the bottom semi-cylinder which supported the head and mask for each dog (h) which attached to the base, the top arches (a) with depth-screws (b) to secure the dog in the appropriate location used as the fiducial marker, the manipulating holding arm (c), and two holding brackets which attached the bottom arch to the top arches and manipulating holding arm. At the end of the holding arm is an adapter which could house different size holding sleeves (d), either for the sleeve (e) for the pointer (f) or for the Steinmann pins. Lastly, g depicts where the mask attaches to the head frame. **(B)** A close up the of the mask design within the Meshmixer software.

To secure each dog in place for use with the BS3D-HF, a headframe was designed in SolidWorks (RRID:SCR_024908) (2023, Dassault Systèmes, United States). Two different headframes were created to accommodate for the different sizes of dogs, one 200 mm and one 300 mm in diameter and depth. The headframes consisted of five major components: the base which secured to the operating table, the bottom semi-cylinder which supported the head and mask for each dog (see creation of the BS3D-HF mask below) which attached to the base, the top arches with depth-screws to secure the dog in the appropriate location, the manipulating holding arm, and two holding brackets which attached the bottom arch to the top arches and manipulating holding arm (Figure 3). The entire headframe was designed so any standard 3D printer could print the headframe.

### 2.6 Creation of the BS3D-HF mask

For each dog, a BS3D-HF mask was created to help secure the dog into the headframe (Figure 3A). The skin and skull STL files were uploaded into Meshmixer. Using the skull STL file, three cylinders with a cone end were placed over each zygomatic arch and occipital protuberance. This was used to help anchor the dog to the headframe (Figure 3). The three cylinders were then connected using arches in such a way to create tension at each cone to help secure the dog in place. Afterwards, the skin STL file was used. Using the select tool, an outline over the surface of the skin from the mandible, to the zygomatic arch, to the maxilla and nasal bone was created bilaterally (Figure 3B). The mask and three-cylinder STL files were then combined, in addition to a bracket that would secure the entire construct to the headframe holding bracket.

### 2.7 Placing the dog images into the headframe

Within Meshmixer, once the mask and three-cylinder STL file unit was created, it along with the skin STL file was placed in the 3D-PDNeuNav headframe. The top arches were then rotated to best accommodate the dog, and the screws of the arches were adjusted to a measured depth that would either go below the surface of the skin STL file by 5 mm, or contact the mask and three-cylinder construct. The angle rotation of the top arches and depth of each screw were recorded for each cadaver as the screws would be used as the fiducial markers in the neuronavigation software.

### 2.8 Printing the BS3D-HF mask and headframe

The mask and headframe STL files were uploaded into Intamsys Intamsuite (V4.2, Shanghai, China) for slicing. PLA filament was used with a 2.85 mm diameter. The printer settings are as follows: layer height: 0.25, shell thickness: 1.0, bottom/top thickness: 1.0, fill density: 20%, print speed: 50 mm/s, printing temp:225°C, bed temp: 60°C, supports: everywhere, overhead angle for support: 45°, fill amount: 15%, platform adhesion: none, flow: 100%. The sliced files were then uploaded to the Instamsys FunMat Pro 410 HT for printing.

### 2.9 Creating the 3D-SCGs

**Figure 4:**
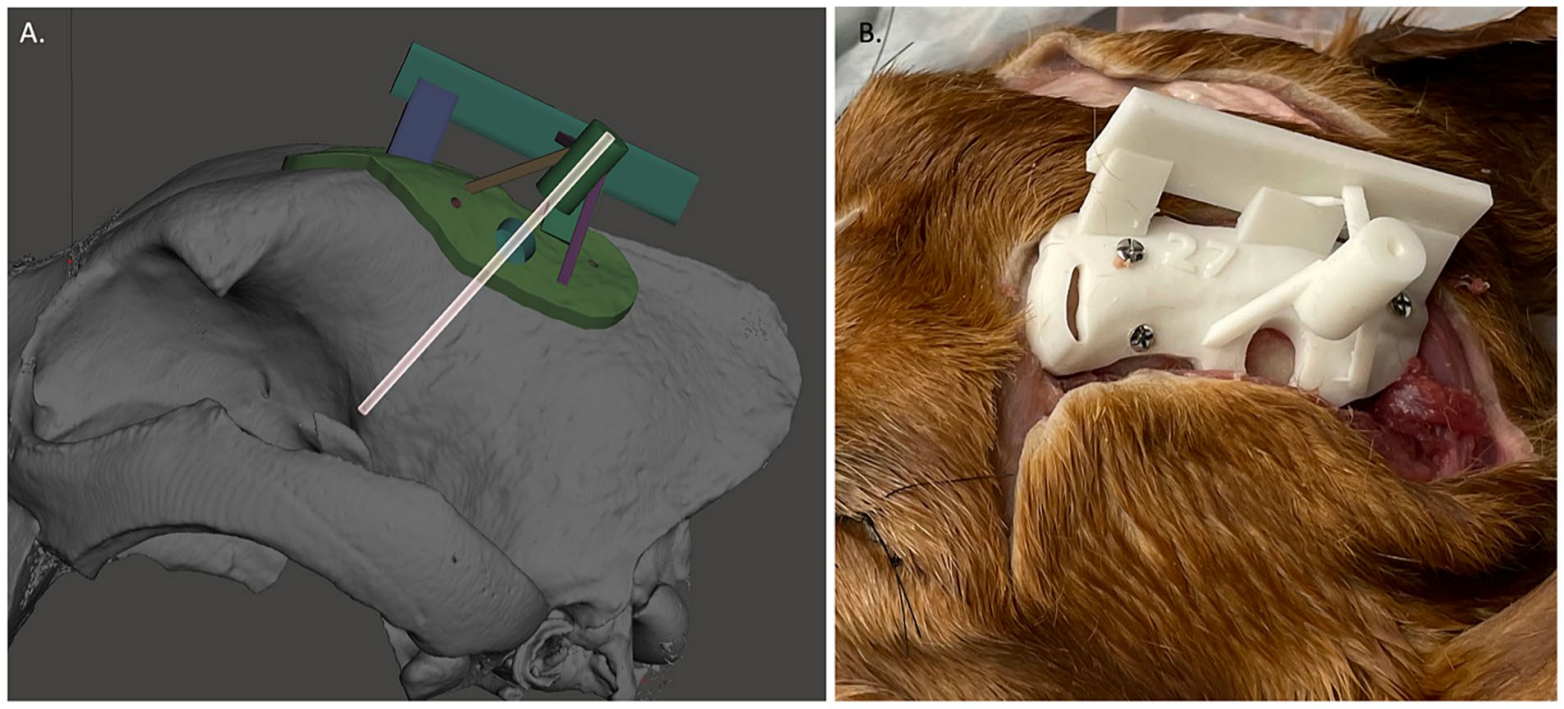
**(A)** Example of 3D-skull contoured guide. The transparent cylinder is shown as an example of how the depth was measured and trajectory was planned. **(B)** Three dimensional-skull contoured guide (3D-SCG) attached to the cadaver.

For each target point, a separate 3D-SCG was made similar to as previously described (9). To summarize and highlight the differences, the skull file for the dog with the ROIs was uploaded into Meshmixer. A 2 mm diameter cylinder was created which extended in an ideal trajectory from the target point to 30 mm beyond the surface of the skull (Figure 4A). Using Meshmixer’s select tool, a region was selected on the skull centered around the cylinder. The region was made large enough to have contact with the skull over multiple curvatures so that it would seat correctly and have room for screw placement, but also adapted to avoid other target point trajectories. The selected region was then extruded 3 mm in thickness and separated from the skull. An 8 mm diameter × 15 mm cylinder was created around the original 2 mm diameter cylinder. Using the Boolean difference tool, a channel in the 8 mm cylinder was created. Supports were then created to connect the footprint of the skull to the cylinder in addition to a cuboid that was above and parallel to midline to ensure proper alignment during the procedure (Figure 4), and the 3D-SCG was exported as an STL file. The STL file was then uploaded into PreForm 3D printing software. Printing thickness was set to 0.05 mm and the auto-setup print function was used for printing. Files were then exported to the Formlabs 3B+ printer and printed using the Formlabs White resin. After printing, 3D-SCGs were then washed in 99% isopropyl alcohol for 20 min, then placed in the Form Cure UV station for 2 h to maximize the resin properties.

### 2.10 Placement of 2 mm diameter double trocar Steinmann pin into target points using 3D-SCG

Given how the 3D-SCGs are placed onto the skull, SP placement into the target point using the 3D-SCGs was performed first. A midline linear incision was made through the skin from the caudal aspect of the frontal fossa to just rostral to the occipital protuberance. The subcutaneous tissue was undermined and reflected ventrally. The temporalis muscle was incised in a curvilinear fashion from the zygomatic process of the frontal bone caudally and a freer elevator was used to undermine the temporalis muscle and reflected ventrally to expose the frontal and parietal bones until the 3D-SCG was able to be placed on the skull (Figure 4B). The 3D-SCG was then placed on the skull and secured with three 2 mm × 7 mm stainless steel screws. The desired depth of the SP was then measured and marked on the SP. A drill was used to advance the SP through the skull and into the brain parenchyma until the measured depth was reached. The 3D-SCG was then removed, dental acrylic was applied around the SP, hardened for 60 s with a UV curing light, then the SP was cut approximately 5 mm above the surface of the skull using pin cutters to minimize interference with other SP placement methods.

### 2.11 Placement of SP into target points using neuronavigation

For each target point, the Brainsight neuronavigation system was used similar as previously described (6). To summarize, the fiducial markers were replaced, and the dog was placed into a surgical head clamp with four skull screws, which was secured to the operating table. The subject tracker was attached to the head clamp along with the articulating arm, and the Polaris Vicra optical position sensor (Northern Digital Inc.) was placed in such a manner the subject tracker and dog could be detected. The CT of the dog was then uploaded into the Brainsight neuronavigation software (V2.5). Following Brainsight protocol, a skin and skull file were created, along with each volumetric ROI for that dog. Afterwards, the trajectory and location for each target point was planned. A live session was then performed. The dog was registered by placing the tip of the neuronavigation pointer into the center of each fiducial marker. Registration was confirmed by using each fiducial marker, bregma, and the occipital protuberance. The neuronavigation pointer was then placed into the articulating arm until the trajectory and target point were aligned. The articulating arm was secured in place, and a 2 mm drill sleeve was placed into the articulating arm. The SP was then measured, depth guard placed, and advanced to the desired depth. Dental acrylic was applied as previously described, the articulating arm was removed, and the SP was cut approximately 5 mm above the surface of the skull using pin cutters. Fiducial markers were then removed.

### 2.12 Placement of SP into target points using BS3D-HF

The process for using BS3D-HF for SP placement has several differences compared to the use of neuronavigation alone. Rather than placing the dog into the head clamp, the dog was initially secured into the previously designed mask and headframe, which is secured to the operating table. The subject tracker was attached to the headframe along with the designed articulating arm (Figure 3), and the Polaris Vicra optical position sensor was again placed in such a manner the subject tracker and dog could be detected. The CT of the dog was then uploaded into the Brainsight neuronavigation software (V2.5). Following Brainsight protocol, a skin and skull file were created, along with each volumetric ROI for that dog. Additionally, the headframe/skin STL file for each dog was uploaded to the Brainsight neuronavigation software and aligned with the skin file created by Brainsight. Afterwards, the trajectory and location for each target point was planned. A live session was then performed. The dog was registered by placing the tip of the neuronavigation pointer into a sleeve which fit onto the end of each depth-screw of the headframe, keeping it parallel and centered with the depth-screw. A total of six points were used for registration. Registration was confirmed by looking at each fiducial marker, the bregma, and the occipital protuberance. The neuronavigation pointer was then placed into the 3D printed articulating arm (Figure 3) until the trajectory and target point were aligned. The articulating arm was secured in place, and a 2 mm drill sleeve was placed into the articulating arm in place of the neuronavigation pointer sleeve (Figure 3). The SP was then measured, marked, and advanced to the desired depth. Dental acrylic was applied as previously described, the articulating arm was removed, and the SP was cut approximately 5 mm above the surface of the skull using pin cutters.

### 2.13 Assessment of each target point

**Figure 5:**
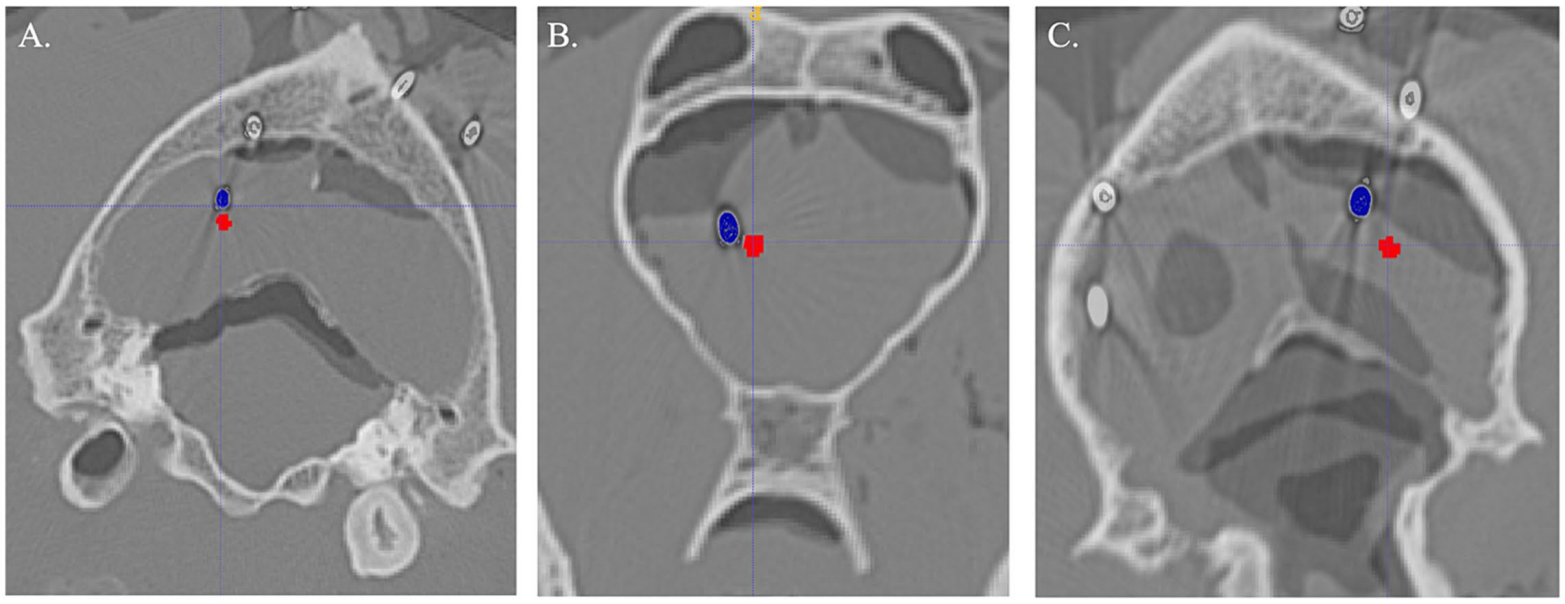
Examples of Steinmann pin (blue) compared to planned target (red) for each method. **(A)** Three-dimensional skull contoured guides. **(B)** Neuronavigation. **(C)** Bright sight combined with a 3D-printed headframe.

After the 30 SPs were placed, CT was repeated as previously described. Initial CTs with ROIs and post SP placement CT files were uploaded into MRIcron (V1.0.20190902) and aligned. Differences between SP placement and target point were recorded in the *X*, *Y* and *Z* direction using the center of the ROI as the point of reference (Figure 5). An average needle placement error was also calculated as follows: √(*X* – *X*′)^2^ + √ (*Y* – *Y*′)^2^ + √ (*Z* – *Z*′)^2^, with the *X*, *Y* and *Z* representing the center planned target point and *X*′, *Y*′ and *Z*′ representing the SP tip location (6).

### 2.14 Statistical analysis

Normal probability plots were inspected to assess the distribution properties of the data, while a Levene’s test was used to evaluate the constant variance assumption. Subsequently, data were summarized as median and range for each method for planned target to the SP placement. Outcomes were compared between methods using linear generalized estimating equations followed by Tukey’s procedure for multiple comparisons. Dog identification was specified as the subject of repetition with exchangeable working correlation between measurements. Agreement between target and pin placement (in pixels) was assessed using Lin’s concordance correlation coefficient. Analysis and graphic generation of data were performed using SAS version 9.4, R statistical software version 4.3.2, and JMP^®^ (Pro 14.0.0) statistical software and reviewed by a statistician (SW). *p*-values <0.05 were considered statistically significant.

## 3 Results

In total, 10 pins were placed for each method, for a total of 30 pins placed. Sixteen pins were placed in the left side, and fourteen in the right. For the 3D-SCG, two pins were placed in the region of the caudate nucleus, three in the hippocampus, two in the midbrain, one in each of the following: occipital lobe, piriform lobe, and thalamus. With neuronavigation, two pins were placed in the region of the caudate nucleus and occipital lobe, three pins were placed in the piriform lobe, and one pin was placed in each of the following sites: hippocampus, midbrain and thalamus. For BS3D-HF three pins were placed in the thalamus, two pins were placed in the hippocampus, occipital lobe, midbrain, and one pin in the caudate nucleus. As each target point was randomly assigned, not every method was used in every dog. Neuronavigation was used in dog 1 (*n* = 3), dog 2 (*n* = 2), dog 3 (*n* = 3), and dog 4 (*n* = 2). The 3D-SCGs were used in dog 1 (*n* = 1), dog 2 (*n* = 3), dog 3 (*n* = 1), dog 4 (*n* = 1) and dog 5 (*n* = 4). The BS3D-HF was used in dog 1 (*n* = 3), dog 2 (*n* = 3), dog 3 (*n* = 1), dog 4 (*n* = 2), and dog 5 (*n* = 1).

**Figure 6:**
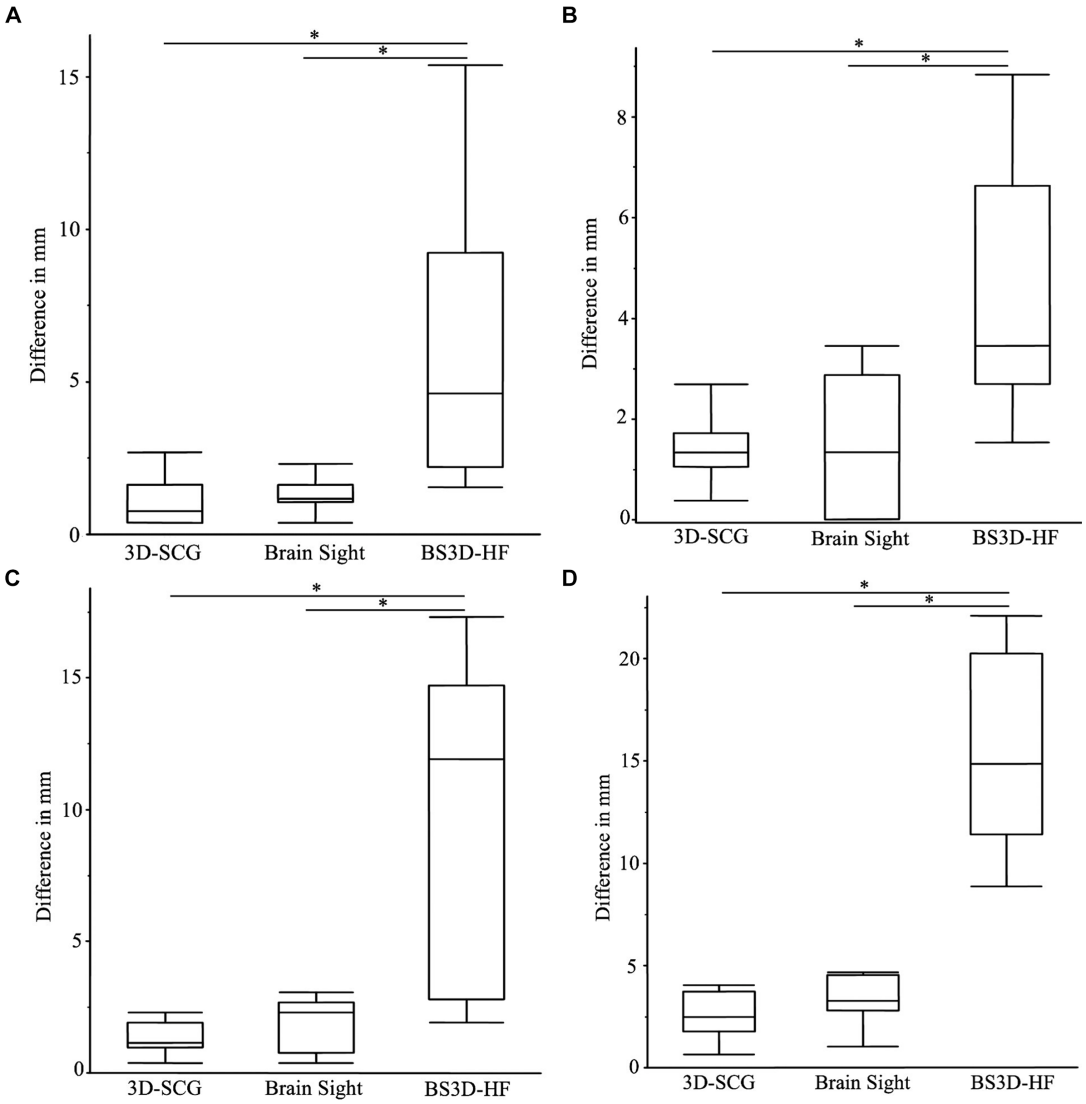
Boxplots of differences between the planned target point and the needle placement in the *X* **(A)**, *Y* **(B)**, *Z* **(C)** directions, and the median deviation **(D)**. The (*) indicates a significant difference between methods of a *p*-value <0.05. In each panel, the box represents the interquartile interval, and the horizontal line within the box the median.

Difference from the planned target point and SP placement for 3D-SCG, neuronavigation, and BS3D-HF are shown in Table 1. When comparing BS3D-HF to 3D-SCG, there was a significant difference in the *X* (*p* = 0.01), *Y* (*p* < 0.0001), and *Z* (p < 0.0001) direction, and for the median deviation (*p* < 0.0001). Additionally, when comparing BS3D-HF to neuronavigation, there was a significant difference in the *X* (*p* = 0.02), *Y* (p < 0.0001), and *Z* (*p* = 0.0003) direction, and for the median deviation (*p* < 0.0001). For comparison of the 3D-SCG to neuronavigation, there was no significant difference in the *X* (*p* = 0.85), *Y* (*p* = 0.82), and *Z* (*p* = 0.72) direction, and for the median deviation (*p* = 0.42). Comparison between the *X*, *Y*, and *Z* direction, and median deviation for each method are shown in Figure 6. Visual representation of the differences is shown in Figure 5.

**Table 1:** Difference between planned target point and Steinmann pin placement for three-dimensional printed skull contoured guides (3D-SCG), neuronavigation, and Brainsight combined with a 3D printed headframe (BS3D-HF).

Type	*X* median difference	*X* range	*Y* median difference	*Y* range	*Z* median difference	*Z* range	Median deviation	Deviation range
3D-SCG	0.76 mm	0.38–2.69	1.34 mm	0.38–2.69	1.15 mm	0.38–2.31	2.48 mm	0.64–4.04
*p*-value	0.98		0.99		1.0		0.99	
Neuronavigation	1.15 mm	0.38–2.31	1.34 mm	0–3.46	2.31 mm	0.38–3.07	3.28 mm	1.04–4.64
*p*-value	0.99		0.98		0.99		0.97	
BS3D-HF	4.61 mm	1.53–15.4	3.34 mm	1.54–8.84	11.9 mm	1.92–17.3	14.8 mm	8.87–22.1
*p*-value	0.73		0.92		0.97		0.87	

## 4 Discussion

When evaluating our primary hypothesis, we were able to confirm that design of BS3D-HF was feasible and was able to be registered within the Brainsight software. However, although both 3D-SCGs and neuronavigation have similar accuracies, the BS3D-HF was significantly less accurate for all points of reference, and thus rejecting our second hypothesis. Although availability and ease of use is important to consider when comparing stereotactic neurosurgical techniques, it is also paramount to assess their accuracy. In veterinary medicine, there is no consensus on minimum accepted accuracies for stereotactic surgery, and this may be variable depending on the neurosurgical indication (9). Reported SBB application accuracy mean and median ranges in veterinary medicine from the planned target are 0.83 to 3.6 mm (2, 5, 7–9, 11, 13, 17). Although the technique with the lowest value would be considered the most accurate, it is not possible to compare studies directly as not every study assesses accuracy the same way, with some studies using only one measurement while other studies used an average of differences to assess accuracy. As direct comparison of SBB methods has never been performed in veterinary medicine, we included two previous established techniques in our study, along with a novel technique (2–17). We did not find a significant difference between SP placement for neuronavigation or 3D-SCGs. Future studies should aim to directly compare novel SBB techniques to previously established methods rather than relying on previous publications. The advantages and disadvantages of each SBB technique must be weighed depending on the specific neurosurgical indication, but improved accuracy for the target is one of the most important factors in deciding which technique to use, and the use of 3D-SCG also allows for bypass of repeated cross-sectional imaging in stage 2 of the workflow.

The standard of care for animals with brain disease has improved over the past decades in large part due to our better understanding of pathology. Although brain biopsy is the gold standard diagnostic test for brain lesions found during advanced imaging, it is not commonly performed due to costs and potential risks, and requirement for anesthesia. Therefore, having a SBB method that is accurate and able to bypass stage 2 of the operative workflow (Figure 1) could increase the number of animals undergoing SBB. From our experience of using frame based, frameless, and 3D-SCG techniques, the 3D-SCGs offer the most straightforward workflow (7, 9). However, the most significant limitation of 3D-SCGs is ensuring the correct planned trajectory once the guide is attached to the skull. If an unexpected error has occurred and the 3D-SCG is not able to seat correctly on the skull, the application accuracy will decrease. We modified our previous 3D-SCG design to include a cuboid along midline, however there is still the concern that during stage 4, surgical refinement is not possible. When using CT for SBB imaging/planning, we have clinically encountered situations, particularly with a biopsy of more superficial intra-axial tumors, in which there is an aberrant or collateral blood vessel occupying or obscuring the majority of the exposed burr-hole craniectomy defect, which subsequently requires either refinement by alteration of the craniectomy defect and/or needle trajectory in order to safely approach the target. This is where the neuronavigation system has an advantage compared to other techniques, as it is not only able to assess the planned trajectory in real time, but also allows for intraoperative alterations. However, as previously stated, additional imaging is required for neuronavigation, the head clamp required for some neuronavigation systems are cumbersome, requiring additional incisions to attach the head frame to the animal, and neuronavigation is subject to accuracy errors due to brain shift, the latter likely being true with all SBB methods, though it has only been investigated with neuronavigation (22).

Although the BS3D-HF was the least accurate technique evaluated, there were several innovations associated with the technique, which can be further improved. First, we were able to design a 3D printed holding arm with a sleeve for the neuronavigation pointer and a sleeve for the SP. The innovative design of the 3D-printed holding arm could be used in a variety of neurosurgical applications with minor modifications. Secondly, another innovation of this study is that we uploaded a 3D design into the Brainsight software for registration, rather than using commercial fiducial markers. This method of using STL files in Brainsight opens the door for other applications such as surgical site planning, and drug delivery devices rather than needing a fiducial marker for registration. A variety of computer-aided design (CAD) files could be used with this method. Finally, the major innovation of this study is our 3D printed head frame for an animal during SBB. We believe that this technique has advantages that make further refinement important. Regardless the preferred SBB technique, any piece of equipment could fail and having an alternative method could be of benefit. Our headframe could be adapted to a variety of SBB techniques to secure the animal in place.

The reasons for the inaccuracy of the BS3D-HFs are likely multi-factorial. A possible general source of measurement error in our study was the use of SPs, which introduce metallic blooming artifact and could have compromised precise determination of the distal pin tip within the target. This could be mitigated through the use a metal artifact correction algorithm, but this was not performed in our study. Additionally, accuracies could have been influenced by target depth selection, as some veterinary SBB studies have suggested that more superficial targets are samples with less accuracy than deeper targets (8, 11), while other studies found no significant associations between needle working length and SBB accuracy (5). Despite that, we suspect the biggest reason for the inaccuracy is due to registration error with the Brainsight software from the BS3D-HF mask not fitting the dog as intended. The procedures were performed in a staged fashion, with initial CT being obtained, and all planning and 3D printing completed prior to performance of the SP placement procedures. Comparing initial and repeated CT, it is clear that autolysis had occurred (Figure 5) not only in the neural parenchyma but also in the muscle. When fitting the mask to the dog, none of the masks fit as well as expected. In the future it would be best to plan the mask solely based on bony structures such as the teeth or skull. We elected to include soft tissue so this method could be more easily adapted to use with MRI rather than CT, but as such, it was not as accurate. An additional possible point of error is that not all the same target points were used in the dogs. Our reasons for randomly assigning target points were that resampling the same target points in the similar/same sized cadavers using different methods was difficult given the technical constraints associated with each of the methods, and could also introduce a learning curve bias into the results on the later methods. However, we had an inhomogeneous group of skulls, thus the skull shape may influence results introducing artificially created difference just by using different skulls shapes for different procedures. Another potential source of error is with the depth-screws of the arches (Figure 3). Within Meshmixer, it is possible to measure the distance to a thousandth of a millimeter. However, when measuring during the procedure with calipers, we could only measure to the nearest millimeter. Furthermore, an alteration in the angle of the calipers might adjust the measurement by only a millimeter but this compounded over each fiducial marker could lead to significant inaccuracies. In the future, designing a device or a depth-screw (Figure 3) which could be measured more precisely would be ideal. Additionally, we added 5 mm of depth to each depth-screw to create a point of pressure on the muscle. Autolysis could have affected this measurement as well. Lastly, the holding arm could have moved when the neuronavigation holding sleeve was replaced with the SP holding sleeve. Although the holding arm was able to easily hold the weight of the neuronavigation pointer (Figure 3A), with enough force, either purposely or accidently, the holding arm could move by small margins even when locked in place. In the future, the holding arm should be fitted with an adapter which can hold both the neuronavigation pointer and the brain biopsy needle to ensure the trajectory has not been altered.

There are limitations to this study. First was the order in which the SPs were placed. Given the limitations of placing the 3D-SCGs, this method was tested first, and had the least deviation from the planned target point. Although trajectory planning with neuronavigation and BS3D-HF was done in such a way to not intersect the 3D-SCG trajectory, it is still possible this could have played a role in 3D-SCG having the lowest deviation. Additionally, the SPs were all placed, then a CT was repeated. It is possible that during placement of additional SPs, the earlier placed SPs were moved. We feel this scenario is unlikely as even without the additional use of the dental acrylic the SPs were firmly fixed. However, as we did not CT between each SP placement, we cannot confirm this, and as this source of error would have had the greatest effect on the 3D-SCGs, it seems less plausible. Another limitation is the repeatability of the techniques. As all SPs were placed by the same investigator, who also designed the 3D prints, and planned each target point, we cannot comment on the accuracy between surgeons. However, SBB techniques are associated with a learning curve and should be tested by each surgeon prior to use on a live animal (23, 24).

In conclusion, although feasible, BS3D-HF should not be used in dogs for SBB without further refinement. Both 3D-SGC and neuronavigation appear to have similar accuracy and are considered appropriate methods for SBB.

## Data availability statement

The original contributions presented in the study are included in the article/Supplementary material, further inquiries can be directed to the corresponding author.

## Ethics statement

The animal study was approved by Virginia Tech Institutional Animal Care and Use Committee. The study was conducted in accordance with the local legislation and institutional requirements.

## Author contributions

RS: Writing – review & editing, Writing – original draft, Visualization, Validation, Supervision, Software, Resources, Project administration, Methodology, Investigation, Funding acquisition, Formal analysis, Data curation, Conceptualization. CH: Writing – review & editing, Writing – original draft, Investigation, Funding acquisition, Conceptualization. RP: Writing – review & editing, Validation. JR: Writing – review & editing, Validation. SW: Writing – review & editing, Formal analysis.

## Supplementary material

SUPPLEMENTARY FIGURE S1 CT imaging artifact associated with manufacturer supplied earbars in a commercially available small animal headframe.
