# Supplementary figures and images for: Comparison of stereotactic brain biopsy techniques in dogs: neuronavigation, 3D-printed guides, and neuronavigation with 3D-printed guides
Source: Front Vet Sci. 2024 Jun 10;11:1406928. doi: 10.3389/fvets.2024.1406928 (PMC11194692; doi:10.3389/fvets.2024.1406928)

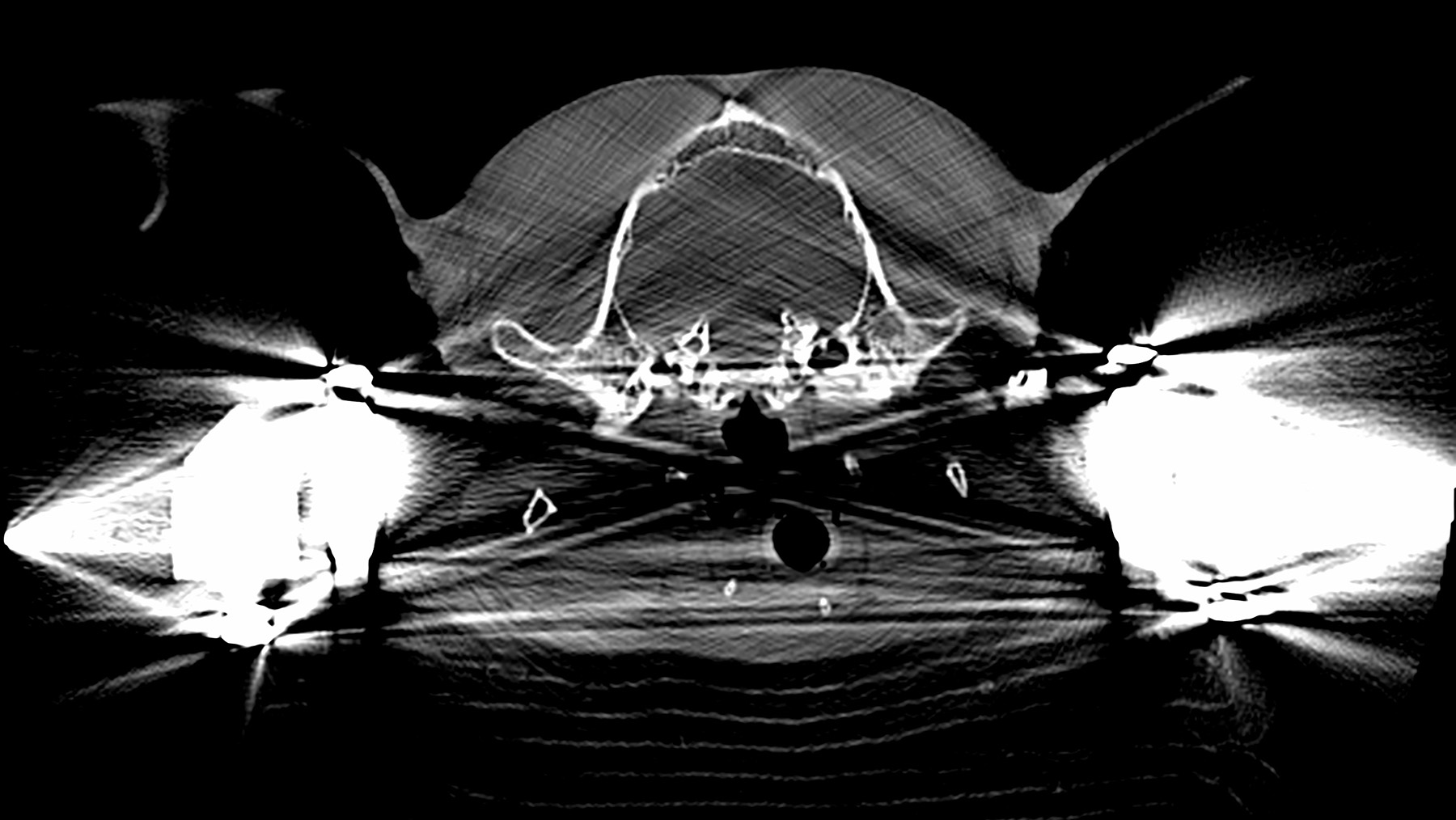

Supplement: SUPPLEMENTARY FIGURE S1 — CT imaging artifact associated with manufacturer supplied earbars in a commercially available small animal headframe. [file Image_1.JPEG]
